# Failure modes and effects analysis for surface‐guided DIBH breast radiotherapy

**DOI:** 10.1002/acm2.13541

**Published:** 2022-02-02

**Authors:** Megan Bright, Ryan D. Foster, Carnell J. Hampton, Justin Ruiz, Benjamin Moeller

**Affiliations:** ^1^ Levine Cancer Institute, Department of Radiation Oncology Atrium Health Cabarrus Concord North Carolina USA; ^2^ Levine Cancer Institute Atrium Health Charlotte North Carolina USA

**Keywords:** SGRT, FMEA, DIBH

## Abstract

Despite breast cancer prevalence and widespread adoption of deep inspiration breath‐hold (DIBH) radiation techniques, few data exist on the error risks related to using surface‐guided (SG) DIBH during breast radiation therapy (RT). Due to the increasingly technical nature of these methods and being a paradigm shift from traditional breast setups/treatments, the associated risk for error is high. Failure modes and effects analysis (FMEA) has been used in identifying risky RT processes yet is time‐consuming to perform. A subset of RT staff and a hospital patient‐safety representative performed FMEA to study SG‐DIBH RT processes. After this group (cohort 1) analyzed these processes, additional scoring data were acquired from RT staff uninvolved in the original FMEA (cohort 2). Cohort 2 received abbreviated FMEA training while using the same process maps that cohort 1 had created, which was done with the goal of validating our results and exploring the feasibility of expedited FMEA training and efficient implementation elsewhere. An extensive review of the SG‐DIBH RT process revealed 57 failure modes in 16 distinct steps. Risks deemed to have the highest priority, large risk priority number (RPN), and severity were addressed with policy changes, checklists, and standardization; of these, most were linked with operator error via manual inputs and verification. Reproducibility results showed that 5% of the average RPN between cohorts 1 and 2 was statistically different. Unexpected associations were noted between RPN and RT staff role; 12% of the physicist and therapist average scores were statistically different. Different levels of FMEA training yielded similar scoring within one RT department, suggesting a time‐savings can be achieved with abbreviated training. Scores between professions, however, yielded significant differences suggesting the importance of involving staff across disciplines.

## 1 INTRODUCTION

Breast cancer is the most common noncutaneous malignancy diagnosed among American women,^1^ of which a significant proportion receive radiation therapy.^2^ Routinely, radiation treatments are delivered to patients with left‐sided cancers using breath‐hold techniques to minimize radiation‐induced cardiac damage.^3^

There has been a paradigm shift in methodology for delivering breast radiotherapy with the emergence of nonionizing surface‐guided radiation therapy (SGRT). Treatment delivery techniques themselves remain relatively simple, often with only two beams tangentially oriented, and yet the complexity of what we ask of the radiation therapy staff has increased dramatically when incorporating SGRT. Rather than using a single or three‐point setup, software is used that models the surface of the patient to ensure an accurate three‐dimensional (3D) setup. SGRT involves using a digital visualization of the patient's surface for set up as well as tracking intrafraction motion. A visible light pattern is projected onto the patient's skin which is used by several cameras to create a 3D rendering of the patient's surface. The rendering is updated in real‐time and displays offsets from either the CT‐generated surface or one created in the software itself.

Failure modes and effects analysis (FMEA) is a risk analysis tool well‐known in the medical physics community since American Association of Physicists in Medicine (AAPM) Task Group (TG) 100 was published in 2016.^4^ FMEA has been used in many areas of radiation oncology, including processes already deemed to be risky such as SBRT/SRS (sometimes in the presence of SGRT) as well as treatment planning system and equipment commissioning, but also to analyze more routine situations such as quality assurance (QA), treatment delays, and chart checks.^5^, ^6^, ^7^, ^8^, ^9^, ^10^, ^11^, ^12^, ^13^, ^14^, ^15^, ^16^, ^17^, ^18^, ^19^, ^20^ Implementation has typically involved a group of experts determining the various opportunities for failure in a given process and then scoring the risks (individually or by consensus) based on standard variables. These variables include the likelihood for occurrence, detectability, and severity; the product of these 3 variables forms a risk priority number (RPN) which is used to compare different potential failures in a process. A threshold is often determined beyond which process improvements or further evaluation would take place.

AAPM TG‐147 recommends investigation of the failure modes of SGRT equipment and the localization process as part of training/acceptance testing.^21^ The rapid implementation of new technology, however, often precludes substantial testing of processes, which perhaps have not even been determined at that early stage. One of the motivations for this work was precisely because of an SGRT‐related error involving bolus that we had not foreseen. Due to the prevalence of breast cancer at a time when the technical standards for treatment are evolving, a risk assessment of established processes seemed prudent. To date, there are no published data concerning the use of FMEA for SGRT‐deep inspiration breath‐hold (DIBH) breast treatments. This project's primary aim was to use FMEA to evaluate SGRT in breast DIBH radiation therapy and report on any findings. Secondary aims included evaluating the scoring differences between groups receiving in‐depth and abbreviated FMEA training as well as differences between staff roles.

## 2 METHODS

### 2.1 Systems and clinical processes at our institution

**FIGURE 1:**
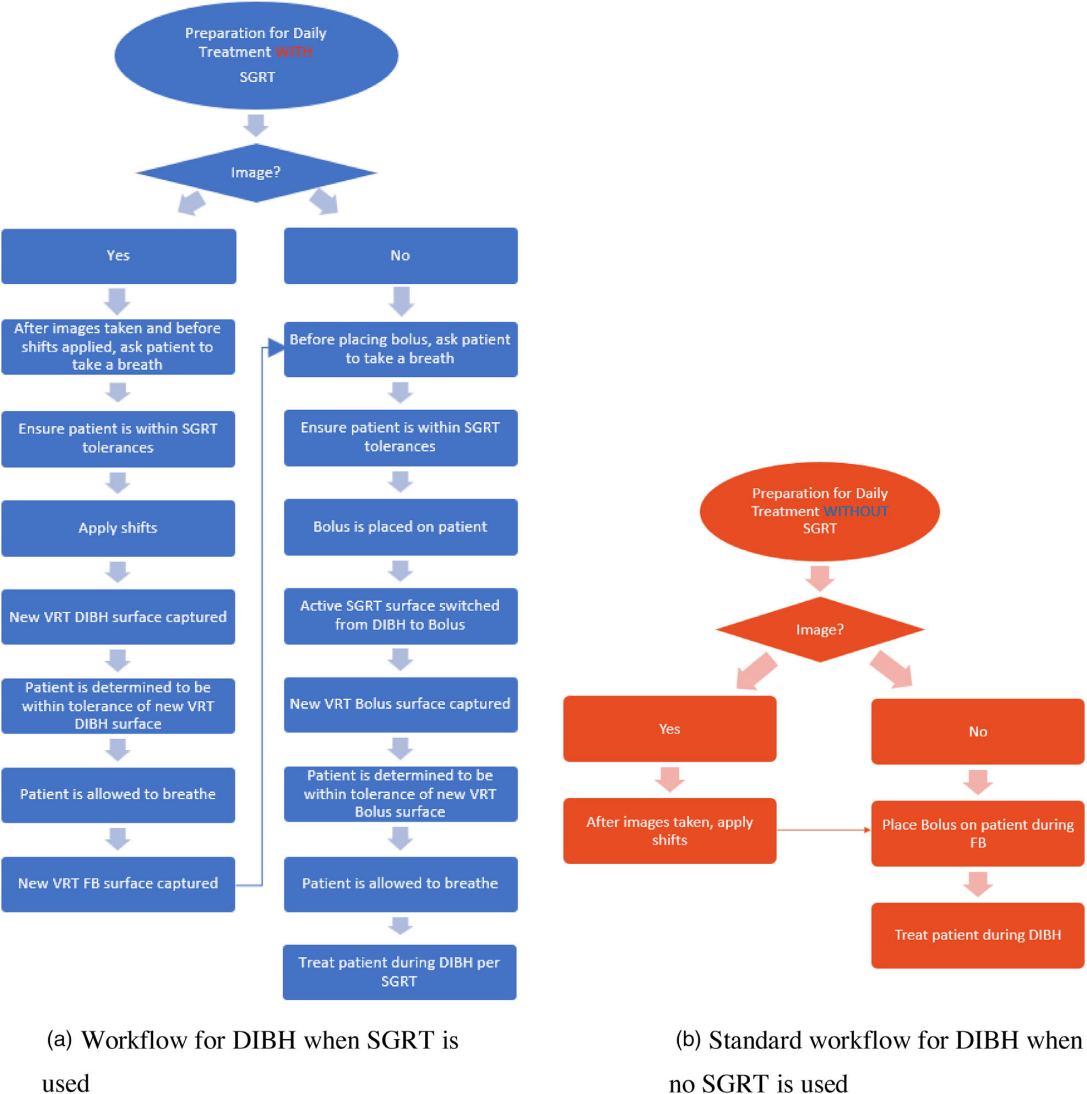
Daily treatment process for deep inspiration breath‐hold (DIBH) with bolus, showing increased complexity with surface‐guided radiation therapy (SGRT) compared to no SGRT. DIBH, deep inspiration breath‐hold; FB‐free breathing; SGRT, surface‐guided radiation therapy; VRT, vision RT generated surface

Voluntary breath‐hold breast treatments, monitored only via video cameras, had been regularly performed at our clinic prior to the installation of our two TrueBeam linear accelerators (Varian Medical Systems, Palo Alto, CA) in 2014 and 2015 equipped with AlignRT SGRT technology (Vision RT, Somerset, NJ). Following the installation, Vision RT's surface guidance cameras and software (AlignRT v.5.0) have instead been utilized to monitor breath‐holds for DIBH treatments. The patient's DICOM data are exported from the treatment planning system (Aria and Eclipse v.13.6, Varian Medical Systems, Palo Alto, CA) and imported into a separate AlignRT offline dosimetry workstation. The physicist checks the fidelity of the imported data and patient creation in the offline workstation; then the therapists pull the patient's data from the offline workstation's mapped network drive to the local drive of the AlignRT workstation at each linac. In our department's clinical SGRT protocol, up to three surfaces are created: free breathing (FB) and DIBH DICOM surfaces, and a temporarily empty bolus surface. The virtual simulation (Vsim) day is designed to be a dry run for the treatment; all initial verification imaging is done that day. First, the patient is breathing normally while the couch position is adjusted to get the patient in tolerance on the DICOM FB surface. Second, the patient's lateral/longitudinal position is fine‐tuned using the DICOM DIBH surface while the patient is in breath‐hold; the vertical position is adjusted only in conjunction with the patient's breath rather than adjusting the couch height. If bolus is used in the treatment plan, the empty bolus surface is used to take a daily surface capture of the patient in breath‐hold with bolus in place using AlignRT; this surface is designated as a vision RT (VRT‐captured) surface. The workflow for a treatment with AlignRT and bolus, including imaging shifts, is shown in Figure 1a and summarized as follows: all within a single breath, the patient is verified to be within tolerance using the DIBH surface, bolus is placed on the patient, the active surface in the AlignRT software is switched from DIBH to bolus, a new VRT reference capture is taken with the bolus in place, and finally the patient is determined to be within tolerance with the newly captured bolus surface. For comparison, Figure 1b illustrates the previously used voluntary breath‐hold process. If at any point the patient is not in tolerance, the bolus is removed, and the patient is set up again as needed with the FB and DIBH surfaces. Weekly MV portal images are taken without bolus during patient breath‐hold; any magnitude shift is applied, with no minimum threshold. If imaging shifts are needed, the shifts would not be applied immediately; instead, during a single breath‐hold, therapists ensure the patient is still within tolerance, the shifts are applied, and a new DIBH VRT reference capture taken. Directly following this process, a new FB VRT surface would be taken and used for patient setup until the next weekly MV imaging session.

Breast tolerances used in AlignRT were 3 mm of translational error and 2 degrees of rotational error. The AlignRT region of interest (ROI) consisted of the treated breast or chest wall, sometimes incorporating the superior supraclavicular region and surrounding chest. The contralateral breast was never included in the DIBH ROI.

### 2.2 FMEA process

The original multidisciplinary group performing the FMEA (Cohort 1) consisted of one radiation oncologist, three therapists, and two physicists. All members of cohort 1 had been regular clinical users of the technology in the same clinic for the same number of years (3), and most had received vendor‐training. Prior to the project commencing, all members received multiple sessions of FMEA training by one of the physicists, who used AAPM TG‐100 as a template,^4^ and a patient safety specialist from the hospital. This safety specialist has a nursing background and obtained dedicated FMEA instruction during an 8‐h didactic course, followed by close support during the facilitation of an FMEA process. Their role was primarily to provide guidance in navigating the FMEA framework, neither to be a data content expert nor participate in the actual risk‐scoring. The FMEA instruction included formalized presentations designed to explain the FMEA concepts as well as informal discussions of example scenarios. For instance, a practice FMEA scenario was examined in an early session: the process of getting ready for work with the failure mode that your alarm clock does not go off. Cohort members worked through the various causes (power outage reset time, alarm not set, etc.) and effects (late to work, omit other steps in morning routine to be on time, etc.). This exercise helped the cohort members enter the FMEA mindset. FMEA training was ongoing; cohort 1 members exchanged information and obtained necessary clarifications during these meetings.

Before the creation of more formalized process maps, the initial meetings were dedicated to brainstorming the SGRT DIBH daily process, first in a big‐picture approach followed by filling in more nuanced details. To limit the scope, the analysis was restricted to the preparation for the Vsim day plus anything that would occur on a typical treatment day; the CT simulation and treatment planning processes were excluded. At these meetings, efforts were made to clarify the process and resolve perception differences, until all group members felt comfortable in their knowledge of both the processes and staff roles in them. This methodology included some discussion of the same process steps on multiple days, between which group members could interview other staff members or be present for the performance of the process. Most differences in understanding revolved around staff in one discipline not knowing details of the process or making incorrect assumptions about what those in another discipline do in each process step; to a lesser degree, there was some disagreement among staff of the same discipline. Process misunderstandings were alleviated in these meetings while variations in scoring were only discussed later, after averages were tallied; this was done to discourage directly influencing group members’ scoring. Notes from these meetings were then organized into a process map, which was created in Microsoft Excel, a logical tool since the clinical process is somewhat linear; using Excel also allowed for efficiency as the scoring could be completed in the same form.

Cohort 1 then determined the failure modes and their current detection methods for each part of the process. This was performed in much the same way as the process mapping, with all members brainstorming possible ways for each process step to fail and what, if any, controls were currently known to be in place.

After the process map and failure mode lists were created, scoring of the severity (S), occurrence (O), and detectability (D) for each failure mode was done independently by each member of the group using blank score copies of the Excel spreadsheet. The main source of scoring guidance came from our health system's patient safety group as well as TG‐100.^4^ Values ranged from 1 to 10 for each category, 10 being the most severe, most likely to occur, and least detectable. RPNs were calculated by multiplying S*O*D for each failure mode. Cohort average RPNs were calculated from the average of each individual's S, O, and D scores. Additional meetings were held to address any clarification pertaining to the scoring guidance.

High severity and risk items were selected for process improvement; the rationale for our choices is explained at length in the Results and Discussion sections. Currently there is a lack of consensus in how these should be chosen; TG‐100, however, recommends that a future task group give guidance on RPN thresholds above which improvement plans should be implemented.^4^ Quality management tools were selected using the guidance in Table 3 in TG‐100, which ranks them by effectiveness.^4^

### 2.3 Comparison with 2nd cohort performing FMEA

A question was raised whether limited FMEA training can provide similar scoring results compared to those individuals who had been extensively trained in this type of risk assessment. Performing FMEA requires a significant time commitment, but substantial risk assessment training for all involved staff‐members is often unrealistic. A shorter FMEA training time investment would be desirable for busy clinics that seek to decrease failures, as well as institutions wanting to include a larger cross section of employees in data collection. Therefore, after the first cycle of scoring, a second group (cohort 2) made up of different staff in the same clinic (three therapists and two physicists) was given abbreviated FMEA training that consisted of only one 30‐min meeting. Cohort 2 received a presentation summarizing the main facets of FMEA: the scoring guidance, the process map format, and the basic clinical DIBH SGRT process, followed by a brief question and answer period. After this presentation, the process map spreadsheet was emailed to cohort 2 members; this was the same scoring spreadsheet used with cohort 1. Cohort 2 had more variation in, but at least a similar level of, SGRT DIBH experience of cohort 1, ranging from 3 to 9 years. One aim was to look for a reasonable reproducibility between the two cohorts in order to provide a degree of validation for the original results. This reproducibility would also provide a reasonable indication that the initial process mapping could feasibly be done by a smaller group, allowing a larger group with minimal FMEA training to perform the scoring. After completing cohort 2′s scoring, these data were both compiled separately from and also combined with the original cohort 1 data to allow for an analysis of the entire dataset and by discipline as well.

## 3 RESULTS

### 3.1 FMEA findings

Sixteen‐process steps resulted in 57 failure modes, and, therefore, a total of 165 causes/effects and possible RPNs. The four highest severity failure modes (representing those with S > 7) were chosen outright to be investigated further. Of these S > 7 modes, only one had an RPN > 92 (mean plus one standard deviation, SD, away); the other three had RPNs ranging 59–77. All three of these high severity failure modes with lower RPNs were “wrong patient” situations. In one of the three failure modes, the wrong patient's surface was used to treat a patient; the D and O scores were low due to many redundant checks ensuring correct patient, including a digital handshake between the SGRT software and treatment console among other non‐SGRT related checks (time‐out with the patient and therapists, checklists verifying patient information, source‐surface distances‐SSDs, imaging). In the second failure mode, FB SSD discrepancies were not verified, leading to a possible wrong patient being treated. Directly after the bolus‐related error in our clinic and during this FMEA, the process was changed so that the DICOM FB surface was used rather than setting FB SSDs, so that this specific failure mode was irrelevant by the end of the project, being nearly identical to the one described previously. The third high severity, lower RPN failure mode was based around using the wrong patient's surface for treatment due to a software glitch that incorrectly bypassed the digital handshake between SGRT and treatment. This mode was based on an actual issue we had seen where software instability (freezing and crashing) caused the incorrect patient to be pulled up. Software updates were implemented during the FMEA that fixed some instabilities and rendered this mode moot; a more general software glitch could have occurred but did not during this project. For these reasons, only one of the top four S > 7 failure modes was included for further scrutiny.

The average RPN data were approximately normally distributed about a mean score of 68, with an SD = 24. Our group originally considered using all high‐scoring failure modes greater than 1 SD away (RPN > 92) but found that 33 was a prohibitively large number of items for which to create action plans. Our hospital safety advisor recommended looking at approximately 10 to avoid being overwhelmed and maximize chances for project completion.

The additional and final selection of failure modes was ultimately determined by scrutinizing the RPN variances across the entire dataset as well as considering redundancies and process overlap in the highest scoring 33 failure modes. Examples of redundancies include previously discriminating between intact breast versus chest wall, and also situations where FB/DIBH surfaces were used instead of DIBH/FB surfaces in the SGRT software; these scenarios were combined due to nearly identical RPNs, and/or any action items would ultimately address both situations. Of the remaining failure modes with an RPN > 92, six were included based on the high variances across scores. For these six failure modes plus the 1 S > 7 mode, action plans were created; the implemented plans address a total of 23 of the 57 failure modes. Table 1 summarizes these seven failure modes, scoring averages, and corrective actions taken to address them. These failure modes were linked with operator error, which can be further classified as manual entry errors (ROI selection and entry of data into AlignRT, including anatomical site, which determines tolerance values) and errors due to lack of attention (choosing incorrect surfaces in AlignRT at treatment and ROI editing).

**TABLE 1:** Cohort 1 failure modes and effects analysis (FMEA) results, showing average O, S, D, and risk priority number (RPN) scores

Process step	Potential failure mode	Potential cause of failure	Occurrence (O)	Potential effects of failure	Severity (S)	Detection methods‐current controls	Detectability (D)	RPN = (O) (S)(D)	Recommended action
Set up with FB DICOM surface	Patient within SGRT tolerance but not positioned correctly	SGRT tolerance too wide due to incorrect site or tolerances adjusted at linac	4.7	Treatment not delivered accurately	7.2	Immobilization, table tolerances, SSDs, physics pretreatment QA, imaging	2.8	95	Establish protocol: physics review FB versus DIBH AP SSDs per patient and adjust tolerances as needed prior to start date Checklist: create Aria Encounter to aid in process
	Patient within SGRT tolerance but not positioned correctly	SGRT fooled: not enough landmarks in ROI	5.0	Treatment not delivered accurately	6.7	Immobilization, table tolerances, SSDs, physics pretreatment QA, imaging	3.3	111	Checklist: create therapist daily Aria Encounter to verify ROI; add to physics weekly encounter to verify ROI
Pull up correct SGRT surface (DICOM or VRT)	Use DICOM mistakenly instead of VRT surface	Imaging form not updated from DICOM; patient moved to other linac	5.7	Treatment not delivered accurately	5.8	Imaging form, imaging	3.0	99	Computerization: map network drive and train therapists how to move files between linacs Checklist: create log to track which patients moved and if images taken
Capture new VRT surface if shifts from imaging	Patient moves or breathes during process	Patient discomfort	5.2	Treatment not delivered accurately	5.3	SGRT, visual inspection of patient, imaging	4.2	115	Establish protocol: change imaging policy to require one therapist to watch patient while the other captures VRT surface; take verification port after all shifts to verify VRT surface
Daily bolus surface captured during DIBH	Patient moves or breathes during process	Patient discomfort	4.7	Treatment not delivered accurately	5.2	SGRT, visual inspection of patient, imaging	4.0	96	Barrier: investigate using rigid bolus to avoid daily VRT captures
	Bolus capture taken with incorrect surface chosen (DIBH or FB)	Labels incorrect or confusing	4.0	Treatment not delivered accurately	5.7	Dates and appearances of SGRT surfaces	4.2	94	Labels: standardize labeling and order of appearance in SGRT; standardize plan order on linac console Checklist: physics to check coincidence of shifts and imaging form changes during weekly QA
	Bolus capture taken with incorrect surface chosen (DIBH or FB)	Lack of attention	4.7	Treatment not delivered accurately	5.5	Dates and appearances of SGRT surfaces	4.3	111	Barrier: investigate using rigid bolus to avoid daily VRT captures Checklist: create therapist daily Aria Encounter to verify ROI

Abbreviations: AP, anterior‐posterior; DIBH, deep inspiration breath‐hold; DICOM, surface created from treatment planning CT; FB, free breathing; QA, quality assurance; ROI, region of interest; SGRT, surface‐guided radiation therapy; SSD, source surface distance; VRT, vision RT generated surface.

### 3.2 Comparison of cohorts 1 and 2

**FIGURE 2:**
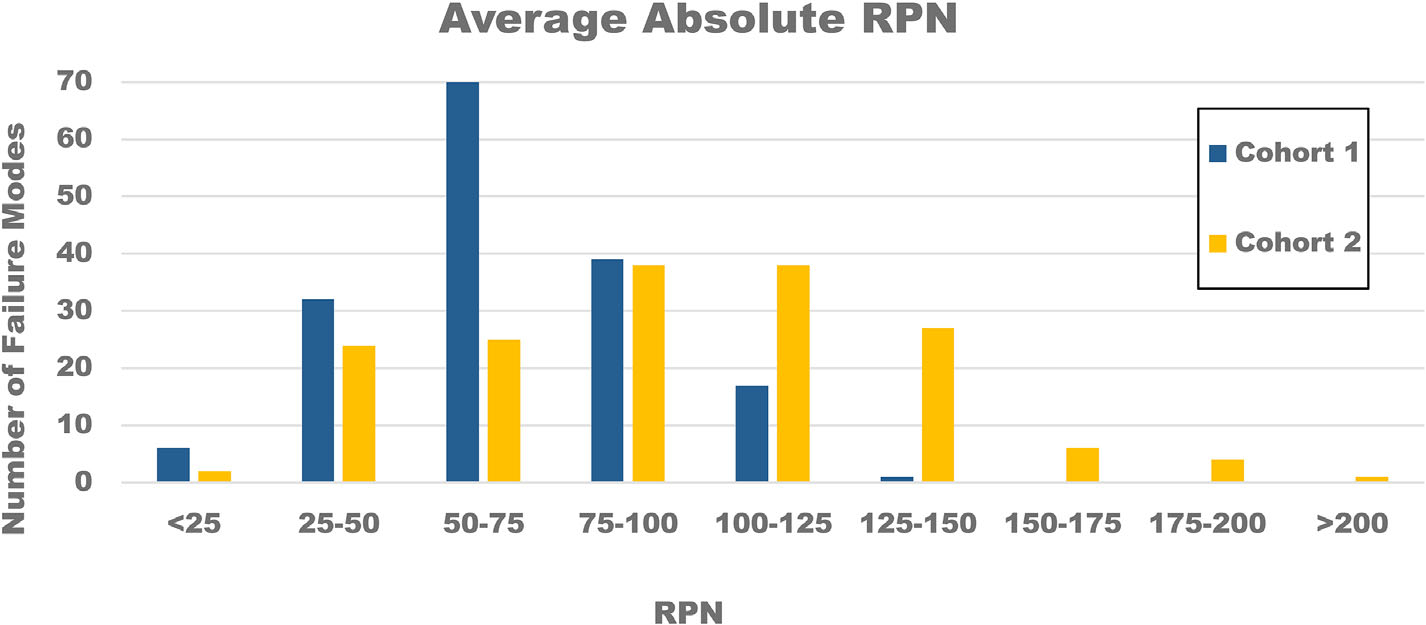
Cohorts 1 and 2 average risk priority number (RPN) scores binned by number of failure modes. Absolute spread of the data, with cohort 2 trending toward greater RPN scores

Figure 2 shows the average RPN differences between cohorts 1 and 2. We see similar Gaussian distributions but with cohort 2 weighted toward larger average RPN values and, therefore, a distinct shift in mean and median; there is also a greater SD among cohort 2 scorers. Cohort 1 has a mean RPN of 68, median 65, SD 24; Cohort 2 has a mean RPN of 95, median 96, SD 39. With the median being nearly equal to the mean, the amount of skew is minimal, and the relative number of outliers is similar on the high and low scoring ends. We would desire to see low variation in the data to suggest that agreement is good between members of each cohort with a minimal number of outliers; the coefficient of variation (CV = SD/mean) is 0.35 for cohort 1 and 0.41 for cohort 2. The larger CV in cohort 2 shows that the variation of this dataset is greater than that of cohort 1, but that both tend to have low overall variation since CV < 1. Using a two‐proportion z‐test, the z score comparing CVs is 0.76 (which is less than the critical z value = 1.96 for a 95% confidence level); we can accept, therefore, that the overall difference in scoring variation between cohorts is not statistically significant.

**FIGURE 3:**
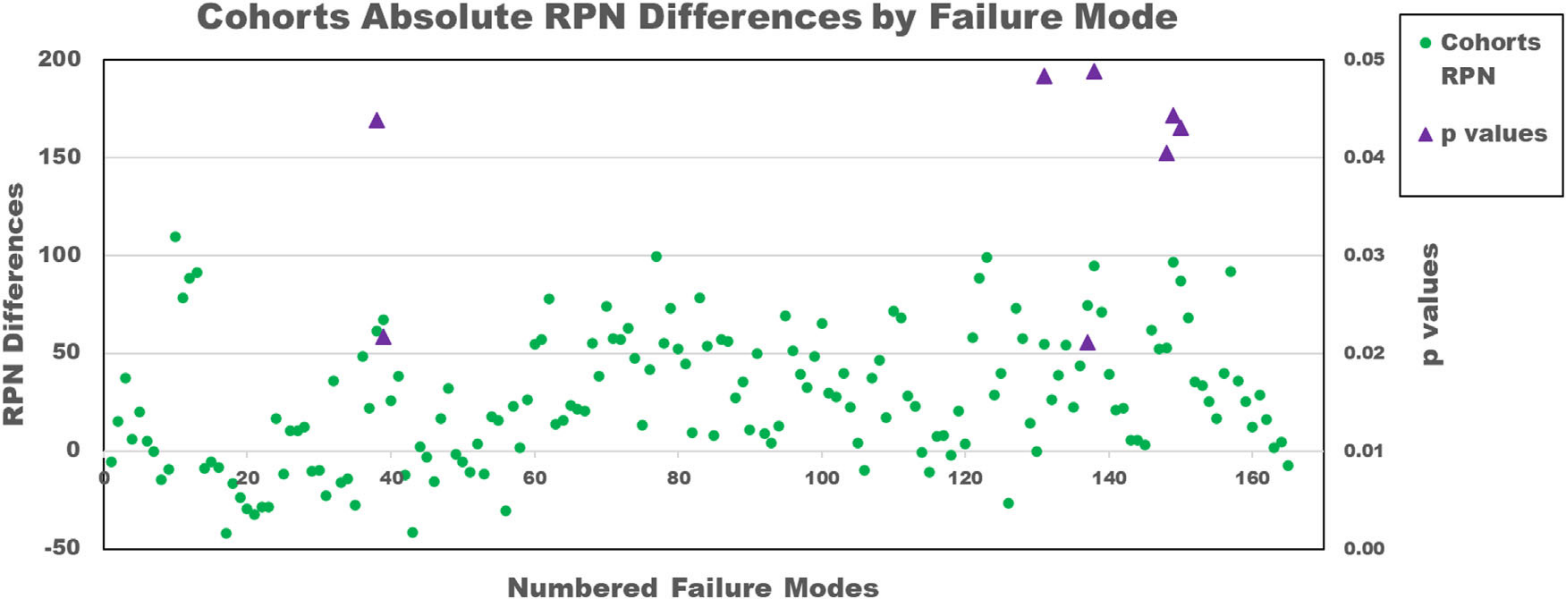
Cohorts 1 and 2 average absolute risk priority number (RPN) differences versus numbered failure mode. Positive RPN differences indicate when cohort 2′s RPN is greater than cohort 1′s RPN. Only significant *p*‐values shown

Figure 3 shows the differences in average RPN scores between the two cohorts for each failure mode and any corresponding significant *p*‐values (*p* < 0.05); the numbered failure modes (1–165) on the x‐axis are based on process chronology only. T‐tests (two‐sided two‐sample assuming unequal variances) were run for each of the 165 individual failure modes using a 0.05 significance level: 5% of failure modes showed statistically significant differences in RPN, 5% for D, 8% for O, and 0% for S between the 2 cohorts.

### 3.3 Scoring comparison by discipline

The slight differences between the cohorts were interesting, so we further investigated by evaluating the data as a function of profession.

**FIGURE 4:**
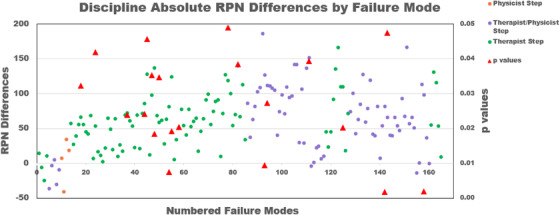
Therapist and physicist average absolute risk priority number (RPN) differences versus numbered failure mode. Positive RPN differences indicate when physicist RPNs are greater than therapist RPNs. Data binned by the discipline involved in the process step. Only significant *p*‐values shown

Figure 4 compares the average RPN differences by discipline versus failure mode between all cohorts 1 and 2 physicists and therapists, excluding the physician “group” as there was only one member; the corresponding significant *p*‐values (*p* < 0.05) are also shown. Two‐sided T‐tests (two‐sample assuming unequal variances) revealed that 12% of RPNs were significantly different: 44% for D, 3% for O, and 19% for S between therapists and physicists. Also in Figure 4, the discipline RPN data were binned by who performed a given process step to determine the existence of any trends; of 16 process steps, one was performed by physicists only, five by therapists and physicists, and 10 by therapists only.

**FIGURE 5:**
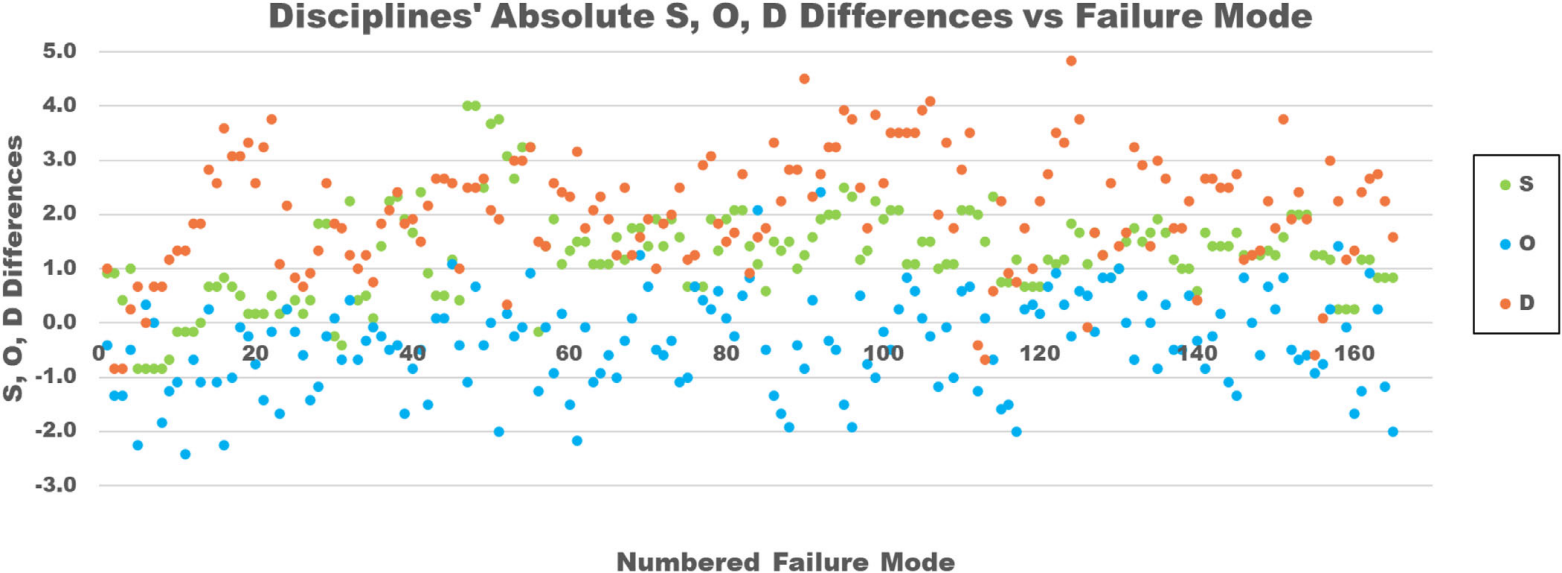
Therapist and physicist average absolute severity (S), occurrence (O), and detectability (D) differences by numbered failure mode. The magnitude of differences between therapists and physicist scores. Positive S, O, and D differences indicate when physicist scores are greater than therapist scores

With the differences seen between physicist and therapist average RPNs, the data were analyzed as separate O, D, and S scores to investigate if one weighed more heavily on the total score than the others. Figure 5 shows absolute differences in occurrence, detectability, and severity by discipline, also for the composite of both cohorts.

## 4 DISCUSSION

### 4.1 FMEA evaluation

In our analysis, the seven failure modes deemed the most severe were those linked with operator error. More specifically, the process steps involving bolus were among those found to have highest priority risks. This is unsurprising considering a bolus‐related error was the driving force behind initiating this FMEA at our institution; this preconceived notion bias could be a potential limitation affecting the results of this work. In an attempt to prevent bolus‐related errors and other failure modes uncovered with FMEA, several established methods for reducing errors were incorporated. The seven failure modes in Table 1 were addressed by using the three most effective types of tools from Table 3 in TG‐100.^4^ Some examples were to add barriers and computerization, establish protocols, add check‐off forms, and standardize labels, as noted in red in Table 1.

Following completion of an initial FMEA, additional work was necessary to map out which of the subsequent processes to focus on for improvement. It would be burdensome to produce an action plan for every failure mode, and that is not the intent according to TG‐100; it is often a compromise in choosing only the highest priority processes to improve with the available resources to act upon them.^7^ As others have done,^10^, ^11^ we have chosen a somewhat arbitrary RPN threshold (1 SD from mean = 92), but more consideration was given to narrowing down our final selections from those 33 failure modes. The variance was calculated for each process/failure mode: those with a high variance were chosen precisely because there was vast disagreement between members of the cohort. In essence, the assumption was made that a lack of agreement of RPN scoring was risky in itself.

The establishment of protocols and checklists was extremely useful for assigning specific tasks to staff. The clinical staff had all been trained in the use of the SGRT software, most by the vendor, so the processes were quite routine. Yet staff reported instances where specific tasks were missed as well as situations where two staff members should have been performing separate but parallel tasks, but instead duplicated the same task, with the other task left undone. Checklists were created within Aria, and therapists now check these items on a daily basis while physicists check them as part of the weekly chart review. According to our FMEA results, reviewing SGRT ROIs is an important aspect of ensuring safe treatments so this became a priority in both physicist and therapist checklists. This cross‐check ensured an independent review of the ROI placement and propagation, as well as factors such as skin tone and whether the VRT surface date was appropriate. Poor choice of ROI placement could cause errors if the patient's surface did not have enough anatomic variation, which is often the case for chest walls with and without breast expanders; ROI propagation can be negatively affected if the gantry is blocking one of the cameras when a new VRT surface is captured, preventing the creation of an adequate 3D rendering. Skin tone, as described to us by the vendor, is a patient‐specific setting that is a function not simply of how dark skin is but how dark the room is. Thus, the skin tone option should reflect how dark the skin appears to the cameras; an extremely dark treatment room may result in changing the setting for all patients to “dark.” As a result of gantry blocking or inadequate skin tone selection, holes may appear on the SGRT surface which would reduce the surface area used to setup and track the patient, possibly causing setup errors or difficulties. Another focus with the new checklists was the correctness of the VRT surface date or whether the DICOM was being used instead. A VRT date would be considered appropriate if it coincided with a date where there were physician‐approved images. There are a variety of situations that can occur where it is possible for the incorrect surface to be chosen. One is that after acquiring a VRT surface 1 week, the next week the DICOM matches portal images better; in this version of software if multiple VRT surfaces had been created, only one appears in clinical mode with the others being automatically inactivated. We would want to discontinue using the VRT surface, but this version of software prevents the ability to delete all VRT surfaces; the workaround we found was to delete the patient entirely from the software and reimport their data to essentially eliminate previous VRT surfaces. This is obviously not ideal with the potential to cause more errors downstream. A newer version of the software allows the DICOM surface or all the VRT surfaces to be inactivated, but with the added potential for error in how VRT surfaces are handled: multiple VRT surfaces can appear in clinical mode, and it is the therapist's responsibility to choose the correct one. How the software handles these types of issues is of extreme importance in order to create processes that try to mitigate software‐specific errors.

Protocols were added that required physicists to review tolerances in a more quantitative way, as one means to prevent errors from occurring during treatment. By performing FB and DIBH CT simulation scans in the same session, we not only obtain both FB and DIBH DICOM surfaces for SGRT but also gain qualitative information on how the patient holds their breath. ROI placement is not only dependent on the treatment site but on where the patient's breathing motion originates from; the fused FB/DIBH CTs can indicate whether patients breathe more from their bellies or their chests. If the patient's chest shows little movement between the two scans yet their belly moves significantly, the ROI may be extended inferiorly to allow for more sensitive gating. The magnitude of the breath‐hold at a reference location (a ball bearing, bb, is placed on mid‐sternum for all our DIBH patients during CT simulation) is also formally noted now as part of our checklist. This bb distance between the two scans is used to determine if our default SGRT tolerances (3 mm) are adequate to detect and track breath‐holds. If the bb breath‐hold distance is less than 3 mm, and the reason is not due to belly‐breathing, the SGRT tolerances may be decreased; QA is then performed, and the likelihood for error decreased, before even getting the patient on the treatment table.

A protocol was also established defining roles for those parallel tasks mentioned before. For example, the therapists know the patient should not move or breathe during the process of capturing a new VRT surface, but exact responsibilities were less obvious. The protocol defines one therapist to be working within the software and the other to monitor the patient during the capture process. While this may seem superfluous as a policy, assumptions in role responsibility could occur with unintended consequences until the roles are discussed and formalized.

Several methods were employed for dealing with possible bolus‐related errors. Therapists’ attention is now drawn to verify the different surfaces via their daily checklist; physicists evaluate these surfaces on a weekly basis. The appearances of the surfaces are reviewed (3D rendering often clearly shows presence of bolus) as well as their labels. Standard order of appearance and labeling of the surfaces attempts to mitigate any errors of choosing the incorrect surface. Previously there had not been standard labels: most often FB was spelled out or FB was used; breath‐hold was spelled out or BH or DIBH was used. The version of SGRT software used for this project had character limits; therefore, BH and FB were agreed upon. Flab bolus is used at our center, but this analysis prompted us to consider rigid bolus instead. Due to the deformable nature of flab bolus, a daily capture for DIBH tracking has been necessary, which increases the likelihood of taking a bolus capture under the incorrect surface or an error occurring elsewhere in the process. Rigid bolus could allow the same SGRT bolus surface to be used daily providing better conformity on the patient, and, therefore, represent a barrier to the further possibility of errors in this part of the process. Several of our other clinics use rigid bolus with great success, mitigating the need to take daily captures; this will continue to be investigated at our center.

### 4.2 Cohort comparisons

The question as to whether abbreviated FMEA training could provide reproducible results was examined next. According to the two‐proportion z‐test, the difference between the cohorts was not statistically significant. There was generally good agreement between cohorts 1 and 2, with only 5% of the RPN scores being statistically different; the largest differences lay with the D and O scores, 5% and 8% respectively. Figure 3 shows the RPN data differences between cohorts and their associated significant *p*‐values. For most of the data, cohort 2′s RPN score is greater than cohort 1′s score, indicating that cohort 2 found the failure modes to be riskier. The fact that cohort 1 had comprehensive group FMEA training and discussions likely influenced D and O scores with the result being less scoring variation; cohort 2 had a slightly larger coefficient of variation than cohort 1, signifying greater variation in scoring, something we might expect from a group with less instruction in FMEA. As noted by a number of authors, training/experience in the technology being studied, in this case SGRT during DIBH, also affects the scoring consistency; there was more variation in SGRT experience across cohort 2 (3–9 years) than cohort 1 (3 years).^17^, ^19^, ^20^, ^22^ This may have affected the scoring variation between cohorts but seems unlikely to be the only cause, since members of both cohorts had used SGRT for DIBH for at least 3 years in the same clinic. The effect of each individual's experience cannot be discounted either, especially since the sample size is small, and the subjectivity of FMEA is well known. Whatever the reasons, the overall agreement between the cohorts was consistent enough to use this model in the future. Our experience here demonstrated that it is feasible for much of the initial work of the FMEA (creating templates and an initial process map) to be done by one person or a small group, which can then be rolled out to a larger group for input and scoring, after some minimal risk assessment/FMEA training. In our own health care system, the goal is to have each clinic review the FMEA using the already‐designed templates. SGRT processes are not currently standardized across our hospital system; it would, therefore, be illuminating to focus additional FMEA on practices that differ the most as well as how the risk scores compare. Reviewing these data could help guide us to less error‐prone methods and allow us to standardize safer processes systemwide.

### 4.3 Analysis of discipline data

In the distribution of data by discipline, 12% of the failure modes had statistically different RPNs when comparing physicists’ and therapists’ scores. Figure 4 shows the RPN data differences between disciplines and their associated significant *p*‐values. In the figure, we see larger RPN differences between disciplines compared to the differences between the cohorts from Figure 3. The scores are also binned by the discipline performing the process step. The one step performed only by physicists involves the QA of all SGRT parameters in the software once the patient data is uploaded; we see that the average RPN differences are relatively low. One example of a therapist/physicist process step is that each group independently reviews the documentation in the patient chart pertaining to SGRT, such as the prescription and our system‐specific image guidance document. Another example of a shared step occurs for each group simultaneously when the bolus surface is captured in the SGRT software; this process step encapsulates numerous micro‐steps and failure modes such as: physicists and therapists verifying the patient is in tolerance before bolus placement, physicists choosing the correct surface in the SGRT software, and therapists ensuring the patient is not moving during and after their bolus placement. The combined therapist/physicist steps and therapist only steps show much more variation and greater overall RPN differences compared to physicist only steps; this suggests that therapists and physicists agree more concerning the risk of physicist‐performed steps than therapist‐performed steps. We also see that the significant *p*‐values lie mainly in the therapist only steps (15% of therapist failure modes, compared to 9% of therapist/physicist modes and 0% for physicist modes), suggesting that physicists find steps that they are not involved in to be riskier than the staff performing them. Since nearly all the RPN differences are positive, physicists score most failure modes as riskier than therapists.

Table 2 describes the seven failure modes that had RPNs that differed the most between physicists and therapists; *p*‐values using the two‐tailed *t*‐test assuming unequal variances for a 0.05 significance level are reported. In all those cases, physicist RPN scores were greater than those of the therapists.

**TABLE 2:** Failure modes associated with the most significant *p*‐values comparing risk priority number (RPN) differences between physicists and therapists

Failure mode	*p*‐Value
Incorrect VRT surface used due to undistinguishable anatomy (example, chest wall)	0.0017
Incorrect VRT surface used due to surface present that should not be (example, bolus surface if bolus was discontinued)	0.0019
Patient moved during the DIBH shift process	0.0094
Incorrect VRT surface used due to surfaces not present when they should be (example, if removed or unapproved so not appearing in treatment mode)	0.020
Incorrect VRT surface used due to incorrect or confusing labels	0.020
Patient not positioned precisely due to wide or incorrectly set tolerances	0.024
Patient not positioned precisely due to poor ROI selection	0.024

Abbreviations: DIBH, deep inspiration breath‐hold; ROI, region of interest; VRT, vision RT generated surface.

Figure 5 shows the O, S, and D score differences between disciplines. While the two groups generally appear to agree on the likelihood for occurrence, the detectability and the severity differ to greater degrees, with physicists being more likely to score a failure mode as more severe and less detectible than the therapists. The O score differences between therapists and physicists are low, with only 3% of the failure modes being statistically different. One possible explanation for the similarity is that staff have the same access to information on errors occurring in both our department and across our network of radiation oncology facilities. Of the three RPN metrics, this is arguably the most objective; the data for clinical errors are readily available and are discussed at quarterly meetings.

The S scores for physicists’ are consistently higher than the therapists’ with 19% of the failure modes being statistically different. Physicists and therapists are clearly placing different valuations on the impact of different failure modes despite having similar levels of experience using SGRT for DIBH. Humans are notoriously poor at assessing risk,^23^, ^24^, ^25^ and the need for risk assessment training is well recognized,^26^ so perhaps a more generalized introduction to risk analysis needs to be emphasized more during FMEA training. Other institutions have created their own scoring system, making the valuation system more explicit and specific to what they are evaluating,^7^, ^18^ which could greatly enhance the precision of FMEA. The scoring system used in this work was more generalized to function in an entire hospital setting. Table 2 in TG‐100 gives specific qualitative and categorical context to the IMRT process (e.g., wrong dose distribution leading to tumor underdose), while our scales were more universally applicable to healthcare (e.g., permanent or major injury). Additionally in some cases, it could be useful to augment the scoring system with new metrics to get to the essence of the process being evaluated.^13^, ^18^

The D scores differ the most, with 44% of the failure modes found to be scored significantly differently between the two groups, with physicists’ scores being much higher in general. This observation in larger D scores possibly based on professional group perceptional differences was also noted when comparing the scores of physicists, radiation oncologists, and neurosurgeons in a Gamma Knife FMEA.^15^ It has been suggested that the D scores, more than the other indices, are based on staff experience level with the technology in question^20^; however, all staff participating in this FMEA had a minimum of 3‐year clinical experience with SGRT for DIBH, the majority in the same clinic. Instead of experience, a likely theory is that this could be due to a lack of understanding or context of QA processes, specifically the significance of the checks, either through not knowing or making false assumptions. A reason could be that many of the QA processes in our department, for both therapists and physicists, were designed by physicists. Therefore, we theorize that physicists have more experience concerning failure mode detection methods. This begs the question of whether the difference in D scores between physicists and therapists would be lessened if more time was spent helping all staff better understood each other's roles in QA and error reduction, knowing not just that QA was performed but the implications of what is, and is not, checked.

A limitation of this project is that the FMEA process requires such an extended timeframe. A new version of the AlignRT software platform was released recently (v.6.2), and several of the FMEA‐evaluated high‐priority processes have changed considerably. With the process maps and failure modes framework in place, however, a clinic is more well‐equipped to quickly assess how changes in software affect our processes.

An additional weakness of using FMEA is the inability to call upon experience when evaluating a relatively new process or technology. As the first radiation oncology center in our system to use SGRT for any patients, there was a dearth of data in our institution's incident learning system pertaining to that technology and thus a lack of empirical information available to help identify possible failure modes. The before‐mentioned bolus error in our clinic was the impetus for performing this evaluation, as this was the first major issue our system had seen since SGRT was initiated. We used what we learned from that error as well as knowledge gleaned from the SGRT community (in the form of meetings, trainings, and journal articles) to create our fault trees and error pathways.

The manner in which a group implements an FMEA process could itself introduce bias; the reliance on a small number (1–3 in our case) of group experts to describe a given process step makes it possible for the results to be skewed by these individuals’ perceptions and experiences. We attempted to mitigate personal bias by discussing the processes with representatives from all pertinent disciplines present, as well as interviewing additional staff members and being present for the clinical performance of the processes.

Despite our best efforts, occasionally when action is taken to address a safety deficiency, the process is instead made riskier. As described in Table 1, there was previously no standardization in SGRT labeling, which led to confusion and mistakes being made. Standard labels were established as a result of the FMEA, but a near‐miss occurred when an incorrect surface was loaded due to the similarity in standardized labels (FB and BH), illustrating an example of FMEA not predicting actual failures—a limitation that has been pointed out by several authors; when a plan of action is finally determined, it has been shown that FMEA has failed to identify failure modes reported in an incident learning system.^7^, ^19^ This caused our group to rethink these labels and in the newer version of software, which does not have strict character limitations, we are using Free Breathing and DIBH. None of this suggests FMEA has no place in radiation therapy, but it must be one of a number of tools used to minimize failures, despite its subjective nature.

## 5 CONCLUSIONS

We have shown that performing FMEA is useful for highlighting potential shortcomings and risks involved when using SGRT in the DIBH process. As a result of the changes implemented from the FMEA, 23 of 57 failure modes were addressed, with the goal of making our DIBH SGRT processes less error‐prone. Various methods were used to mitigate error, including checklists, standardization, protocols, barriers, and computerization. Despite the increase in technology, many of the processes still require manual inputs or checks; the highest‐scoring failure modes reflected lapses in these areas. For a large network of clinics, it is feasible for a small group to do the initial work of designing the process trees and creating the scoring guide, then pass the scoring onto a larger group. Dedicated FMEA training, however, may be preferable to ensure understanding of the process if one goal is to maximize scoring consistency. Additional training in generalized risk assessment may be desirable. The larger scoring differences seen by discipline compared to FMEA training level highlights the importance of discussions pertaining not only to risk but to understanding the role each of us plays in ensuring high‐quality, error‐free care. Centers starting new SGRT DIBH programs may find value in using our experience as a guide, while experienced centers may be prompted to look for gaps in their own established programs.

## CONFLICT OF INTEREST

The authors declare that there is no conflict of interest that could be perceived as prejudicing the impartiality of the research reported.

## AUTHOR CONTRIBUTION

Megan Bright conceived of, designed, acquired, and analyzed the data, wrote drafts, approved for publication, and agree to be responsible for the content of the manuscript. Carnell Hampton, Ryan Foster, Justin Ruiz, and Benjamin Moeller contributed to the acquisition and interpretation of data, revisions of drafts, approval for publication, and agree to be responsible for the content of the manuscript.

## Supporting information

Supporting Information
