# Supplementary material for: Failure modes and effects analysis for surface‐guided DIBH breast radiotherapy
Source: J Appl Clin Med Phys. 2022 Feb 2;23(4):e13541. doi: 10.1002/acm2.13541 (PMC8992938; doi:10.1002/acm2.13541)
Supplement: Supplementary file 1 — Supporting Information [file ACM2-23-e13541-s001.docx]

**Failure Modes and Effects Analysis for Surface Guided DIBH Breast Radiotherapy**

Megan Bright, MS^1^

Staff Medical Physicist

Corresponding Author

[megan.bright@atriumhealth.org](mailto:megan.bright@atriumhealth.org)

**FMEA for SGRT Breast DIBH**

Ryan D. Foster, Ph.D.^1^

Chief Medical Physicist

[ryan.foster@atriumhealth.org](mailto:ryan.foster@atriumhealth.org)

Carnell J. Hampton, Ph.D.^2^

Assistant Vice President-Physics

[carnell.hampton@atriumhealth.org](mailto:carnell.hampton@atriumhealth.org)

Justin Ruiz, MS^1^

Staff Medical Physicist

[justin.ruiz@atriumhealth.org](mailto:justin.ruiz@atriumhealth.org)

Benjamin Moeller, MD, Ph.D.^1^

Medical Director

[benjamin.moeller@atriumhealth.org](mailto:benjamin.moeller@atriumhealth.org)

^1^Levine Cancer Institute Department of Radiation Oncology, Atrium Health Cabarrus, 920 Church Street North, Concord, North Carolina 28025

^2^Levine Cancer Institute, Atrium Health, 1021 Morehead Medical Drive, Charlotte, North Carolina 28204

**Author contribution statement**

Megan Bright conceived of, designed, acquired and analyzed data, wrote drafts, approved for publication, and agree to be responsible for the content of the manuscript. Carnell Hampton, Ryan Foster, Justin Ruiz, and Benjamin Moeller contributed to the acquisition and interpretation of data, revisions of drafts, approval for publication, and agree to be responsible for the content of the manuscript.

**Acknowledgements**

This work could not have been done without the help of our fantastic therapists who volunteered their time and expertise in this project: Jennifer Konkle, Jessica VanMarter, Andrew Carte, Emily Hartleben, Katherine Overcash, and Lindsay Stallings. In addition, a special thanks to Cheryl Weiner, our superlative hospital patient safety representative.
